# Antegrade enteral stenting for afferent loop syndrome using the double-guidewire technique via endoscopic ultrasound-guided hepaticogastrostomy

**DOI:** 10.1055/a-2740-3913

**Published:** 2025-11-27

**Authors:** Yuki Fujii, Kazuyuki Matsumoto, Daisuke Uchida, Shigeru Horiguchi, Koichiro Tsutsumi, Motoyuki Otsuka

**Affiliations:** 1 199491 Department of Endoscopy, Okayama University Hospital, Okayama, Japan; 2 Department of Gastroenterology and Hepatology, Okayama University Hospital, Okayama, Japan

As endoscopic treatments for afferent loop syndrome (ALS), endoscopic enteral stent placement (EESP) and endoscopic ultrasound-guided entero-enterostomy (EUS-EE) have been mainly reported 1 2 . The double-guidewire technique (DGT) has been reported to facilitate stent placement during endoscopic ultrasound-guided hepaticogastrostomy (EUS-HGS) by making the guidewire angle obtuse 3 . We report a case of malignant ALS in which antegrade EESP was achieved via the EUS-HGS route using the DGT.

A 67-year-old woman underwent distal gastrectomy with Roux-en-Y reconstruction for advanced gastric cancer. During adjuvant chemotherapy, she developed abdominal pain and jaundice. Contrast-enhanced computed tomography (CE-CT) revealed distal bile duct and duodenal stenosis due to recurrent peritoneal dissemination ( Fig. 1 ). She was diagnosed with ALS and obstructive jaundice and was transferred to our hospital. Initially, EESP using balloon-assisted endoscopy was attempted; however, adhesions prevented access to the obstructed segment ( Fig. 2 **a, b** ). EUS-EE was difficult because of the considerable distance between the remnant stomach and the afferent loop. Therefore, EUS-HGS was planned with simultaneous intestinal drainage if technically feasible. The B3 branch was punctured using a 19-gauge needle, and contrast injection revealed obstruction of the distal bile duct and the afferent loop ( Fig. 3 **a, b** ). Marked angulation of the afferent loop presented a high risk of stent kinking or perforation with metal stent placement; therefore, plastic stents (PSs) were selected. Using a UDLC (Piolax Medical, Kanagawa, Japan), DGT with an additional stiff 0.035-inch guidewire made the guidewire angle obtuse, allowing the placement of two PSs in the afferent loop ( Fig. 3 **c, d** , Fig. 4 **a, b** , Video 1 ). Subsequently, uncovered and partially covered self-expandable metal stents were placed in the distal bile duct and the EUS-HGS site, respectively, using the DGT ( Fig. 3 **e** ). Follow-up CE-CT showed resolution of the afferent loop and bile duct dilatation, with laboratory improvement ( Fig. 5 ). The patient was discharged on day 8 postoperatively.

**Fig. 1:**
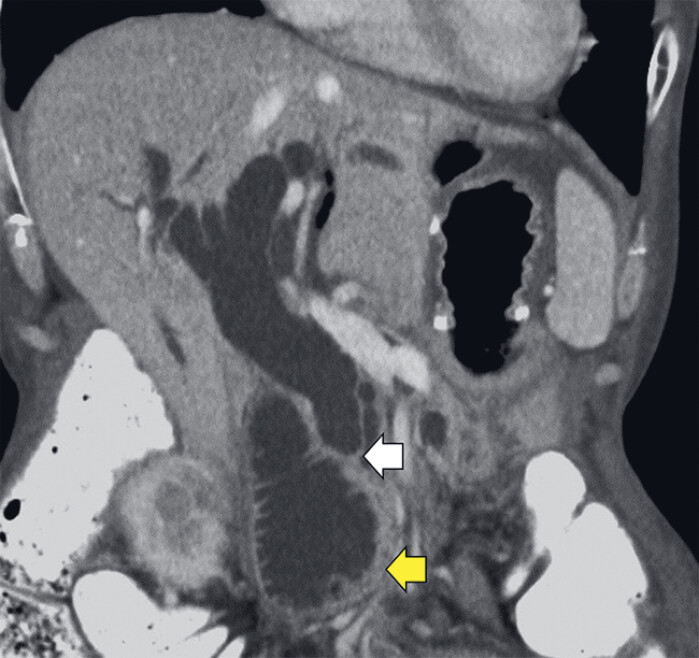
Contrast-enhanced computed tomography (CE-CT; coronal view) demonstrates peritoneal dissemination-induced stenosis of the distal bile duct (white arrow) and the third portion of the duodenum (yellow arrow), with dilatation of the bile duct and the afferent loop. The dilated afferent loop is situated at a considerable distance from the remnant stomach.

**Fig. 2:**
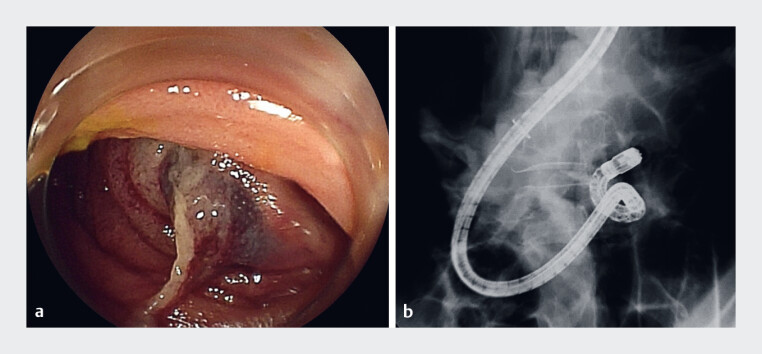
Balloon-assisted endoscopy was attempted; however, intra-abdominal adhesions prevented access to the obstructed segment. **a** Endoscopy image and **b** X-ray image.

**Fig. 3:**
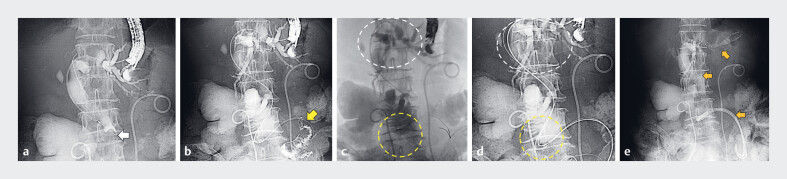
**a** The B3 branch was punctured from the remnant stomach, and cholangiography reveals an obstruction in the distal bile duct near the papilla (white arrow). **b** Contrast injection into the afferent loop reveals the stricture (yellow arrow), and the guidewire was successfully advanced through it. **c, d** A single guidewire is placed across the strictures of the distal bile duct and the afferent loop ( **c** ). In the double-guidewire technique, a stiff 0.035-inch guidewire was added to the 0.025-inch guidewire using a UDLC, which blunted the guidewire angulation ( **d** ). White and yellow dotted circles highlight the areas where the guidewire angle was markedly blunted. **e** Two double-pigtail plastic stents, an uncovered self-expandable metal stent, and a partially covered self-expandable metal stent are placed at the afferent loop stricture, distal bile duct stricture, and hepaticogastrostomy site, respectively (orange arrows).

**Fig. 4:**
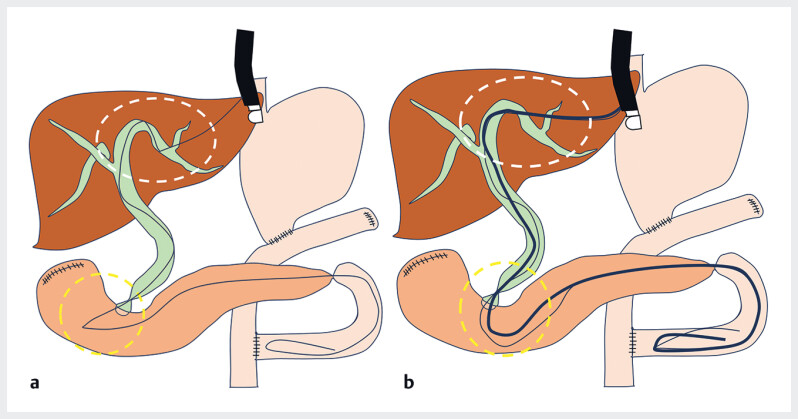
**a, b** A single guidewire is left in place via the hepaticogastrostomy route ( **a** ). In the double-guidewire technique, placement of two guidewires, including a stiff 0.035-inch guidewire, blunts the acute angle and stabilizes the axis, thereby facilitating device delivery ( **b** ). The two dotted circles highlight the areas where the guidewire angle was markedly blunted.

**Fig. 5:**
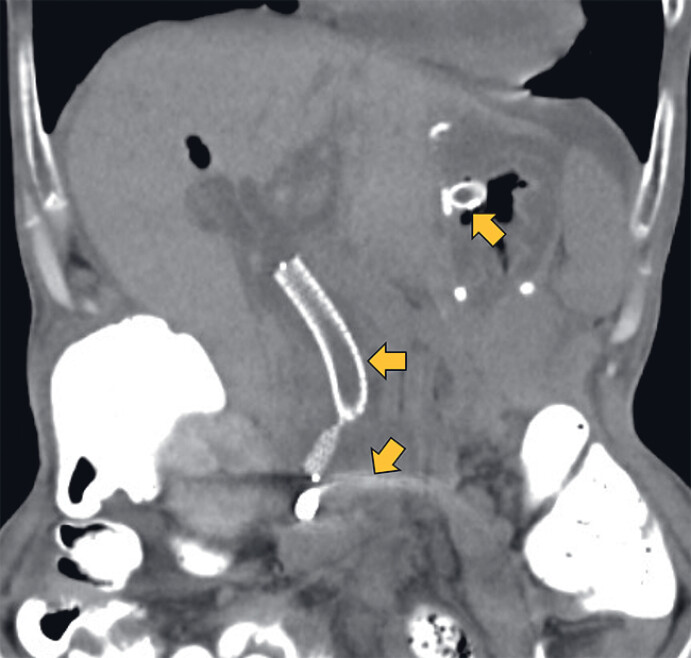
Follow-up computed tomography demonstrates the disappearance of the afferent loop and bile duct dilatation. Orange arrows indicate the stents.

Video highlights the effect of DGT on straightening the axis, improving device deliverability. DGT, double-guidewire technique.Video 1

Endoscopy_UCTN_Code_TTT_1AS_2AB

## Notes

Endoscopy E-Videos https://eref.thieme.de/e-videos
*E-Videos* is an open access online section of the journal *Endoscopy* , reporting on interesting cases and new techniques in gastroenterological endoscopy. All papers include a high-quality video and are published with a Creative Commons CC-BY license. Endoscopy E-Videos qualify for HINARI discounts and waivers and eligibility is automatically checked during the submission process. We grant 100% waivers to articles whose corresponding authors are based in Group A countries and 50% waivers to those who are based in Group B countries as classified by Research4Life (see: https://www.research4life.org/access/eligibility/ ). This section has its own submission website at https://mc.manuscriptcentral.com/e-videos .
